# *In situ*-prepared composite materials of PEDOT: PSS buffer layer-metal nanoparticles and their application to organic solar cells

**DOI:** 10.1186/1556-276X-7-641

**Published:** 2012-11-23

**Authors:** Sungho Woo, Jae Hoon Jeong, Hong Kun Lyu, Yoon Soo Han, Youngkyoo Kim

**Affiliations:** 1 Green Energy Research Division, Daegu Gyeongbuk Institute of Science and Technology (DGIST), Daegu, 711-873, South Korea; 2 Organic Nanoelectronics Laboratory, Department of Chemical Engineering, Kyungpook National University, Daegu, 702-701, South Korea; 3 Department of Advanced Energy Material Science and Engineering, Catholic University of Daegu, Gyeongbuk, 712-702, South Korea

**Keywords:** *In situ* preparation, PEDOT:PSS-metal NPs, Enhanced light absorption, Organic solar cells

## Abstract

We report an enhancement in the efficiency of organic solar cells via the incorporation of gold (Au) or silver (Ag) nanoparticles (NPs) in the hole-transporting buffer layer of poly(3,4-ethylenedioxythiophene):poly(styrenesulfonate) (PEDOT:PSS), which was formed on an indium tin oxide (ITO) surface by the spin-coating of PEDOT:PSS-Au or Ag NPs composite solution. The composite solution was synthesized by a simple *in situ* preparation method which involved the reduction of chloroauric acid (HAuCl_4_) or silver nitrate (AgNO_3_) with sodium borohydride (NaBH_4_) solution in the presence of aqueous PEDOT:PSS media. The NPs were well dispersed in the PEDOT:PSS media and showed a characteristic absorption peak due to the surface plasmon resonance effect. Organic solar cells with the structure of ITO/PEDOT:PSS-Au, Ag NPs/poly(3-hexylthiophene):[6,6]-phenyl-C61-butyric acid methyl ester (P3HT:PCBM)/LiF/Al exhibited an 8% improvement in their power conversion efficiency mainly due to the enlarged surface roughness of the PEDOT:PSS, which lead to an improvement in the charge collection and ultimately improvements in the short-circuit current density and fill factor.

## Background

Organic solar cells (OSCs) have recently been studied intensively for use in the next-generation photovoltaic devices due to their lower material cost, large area, flexibility, and simple solution processability [1,2]. Although their power conversion efficiency (PCE) levels have been improved to 5% based on the bulk heterojunction of poly(3-hexylthiophene) (P3HT) and [6,6]-phenyl-C61-butyric acid methyl ester (PCBM), further improvement of up to 10% is required for successful commercialization [3,4]. The main drawback of OPV is the limitation of the exciton diffusion length to about 10 to approximately 20 nm and the poor charge transport property. Moreover, the difficulty of increasing the active layer thickness leads to insufficient absorption of incident light [5,6]. To improve light absorption in the organic photoactive layer, several methods, including the introduction of new low-band-gap polymers [7], the use of an inorganic optical spacer between active layer and the metal electrode [8], and the application of surface plasmons (SPs) based on metal nanoparticles (NPs) incorporated in the active layer or deposited onto an indium tin oxide (ITO) electrode [9,10] have been reported. Among these methods, plasmonic enhancement is considered to be one of the best approaches due to its simple process, effectiveness, and controllability of the plasmon absorption wavelength by adjusting the particle size, shape, and compositions. SPs are collective surface oscillations of conduction electrons within metal nanostructures that tend to trap optical waves near their interface between the metal and a dielectric medium under electromagnetic field excitation. They can create strong near-field electromagnetic fields and far-field propagating waves, which can be used for the enhancement of the light absorption and photocurrent of OSCs [11]. Three methods using the SP phenomena of metal NPs in OSCs have been reported, but they all have some drawbacks. The first involves the use of metal NPs incorporated into the photoactive layer, but in this case the PCE can be restricted by exciton quenching with nonradiative energy transfer and the differences between the electronic properties of the metal NPs and the conjugated photoactive molecules [12,13]. Recently, however, several reports on the successful application of Au or Ag NPs into a photoactive layer have been published, demonstrating the enhancement of optical and electrical properties based on SPs as well as other additional effects (e.g., increased surface roughness and balanced charge mobilities) [14-17]. The second method involves the formation of a metal NP layer between the ITO electrode and the poly 3,4-ethylenedioxy-thiophene:polystyrene sulfonate (PEDOT:PSS) buffer layer, which typically requires an extra deposition process such as the vacuum deposition of a metal nanolayer with a sequential thermal treatment or the spin-coating of a metal NP precursor solutions [18,19]. The third method relies on a metal-NP-incorporated PEDOT:PSS layer [20-22]. Both the first and third methods require a two-step experimental process, including the aqueous synthesis of metal NPs using organic capping agents such as poly(ethylene glycol) (PEG) or poly(vinyl pyrrolidone) (PVP) and the mixing/dispersion of synthesized metal NPs with a photoactive or PEDOT:PSS solution.

Here, we suggest a simpler method to introduce metal NPs into PEDOT:PSS without the need for organic capping agents or an additional mixing process via *in situ*-prepared composite materials of PEDOT:PSS-metal NPs in which Au or Ag NPs are stably incorporated. We also investigate the effects of metal NPs on the device properties as to whether they act as SPs or another mechanism.

## Methods

### Synthesis of PEDOT:PSS-Au or Ag NPs composite materials

**Figure 1:**
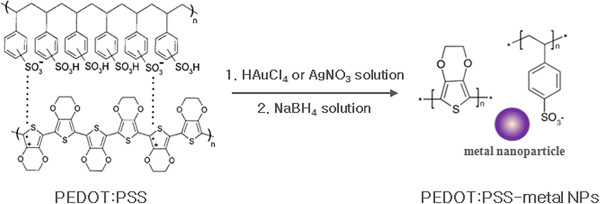
***In situ *preparation of PEDOT:PSS-metal NP composite solutions.**

We developed a novel *in situ* means of preparing stabilized Au or Ag NPs by the reduction of chloroauric acid (HAuCl_4_) or silver nitrate (AgNO_3_) with a sodium borohydride (NaBH_4_) solution in the presence of aqueous PEDOT:PSS media. Briefly, an aqueous solution of 5 mM HAuCl_4_ or AgNO_3_ was added to 5 ml of PEDOT:PSS solution (Clevios PH500, Heraeus Clevios GmbH, Leverkusen, Germany) under vigorous stirring. A 10 mM of NaBH_4_ was then injected into the stirred mixture very slowly (see Figure 1). The AgNO_3_ (≥99.5%), HAuCl_4_ (≥99.9%), and NaBH_4_ (≥ 99%) used here were all purchased from Sigma-Aldrich Co., Ltd., St. Louis, MO, USA.

### Device fabrication

**Figure 2:**
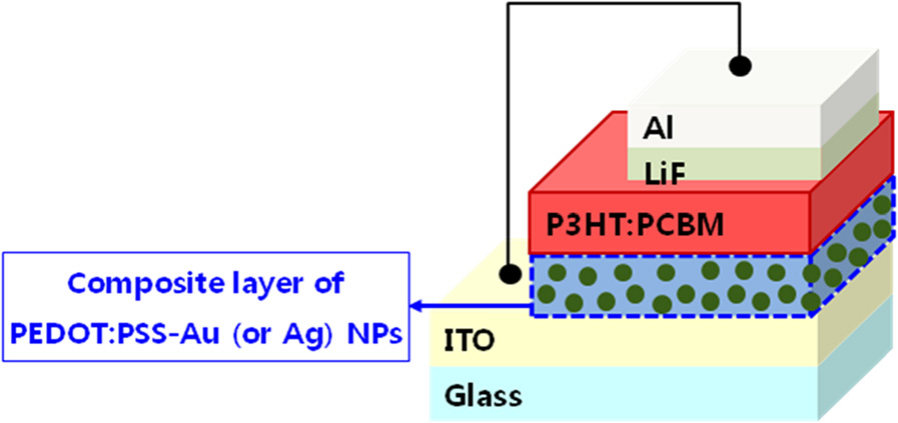
Structure of the OSC devices.

In order to investigate the effects of Au or Ag NPs in PEDOT:PSS buffer layer, we prepared three types of OSC devices with pristine PEDOT:PSS, with PEDOT:PSS-Au NPs, and with PEDOT:PSS-Ag NPs as a hole-conducting buffer layer. The device configuration was glass/ITO (10 Ω/sq)/buffer layer (40 nm)/P3HT:PCBM (100 nm)/LiF (0.5 nm)/Al (150 nm), as shown in Figure 2. To fabricate the OSC devices, a patterned ITO glass was cleaned by sequential treatments with de-ionized water, acetone, and isopropanol in an ultrasonic bath for approximately 10 min. The buffer layer, PEDOT:PSS, was spin-coated on the ITO/glass substrate and thermally dried at 140°C for 30 min. The photoactive layer was a mixed solution of P3HT (Rieke Metals Inc., Lincoln, NE, USA)/PCBM (Nano-C, Westwood, MA, USA) blend (1:0.9) dissolved in 1,2-dichlorobenzene. The active layer was spin-coated on top of a buffer layer and dried on a hot plate for 40 min at 50°C to achieve a thickness of 100 nm. Finally, a cathode layer of LiF/Al was deposited by thermal evaporation under a vacuum of 1.0 × 10^−7^ Torr. A post-annealing process was done at 140°C for 20 min in a N_2_-filled glove box system. The active area of the devices was defined as 9 mm^2^ by a shadow mask.

## Results and discussion

Au and Ag NPs are among the most widely used nanomaterials in biological and electronic applications. Such applications, however, require these particles mostly to be water-dispersible and/or suspended in water without a loss of their physical or chemical properties over long periods of time. To obtain stable NPs in media with a high ionic strength (water), organic capping stabilizers such as PEG, PVP, cetyltrimethylammonium bromide, or sodium dodecylsulfate, which cannot easily be removed during processing for further applications, are required. While synthetic methods of NPs in organic media were reported by several groups with a well-defined size and shape, they are water-immiscible and have limited application range [23]. Recently, research on combining these NPs with PEDOT:PSS has been conducted in an effort to prepare hybrid materials for use as a surface plasmon source [20-22].

**Figure 3:**
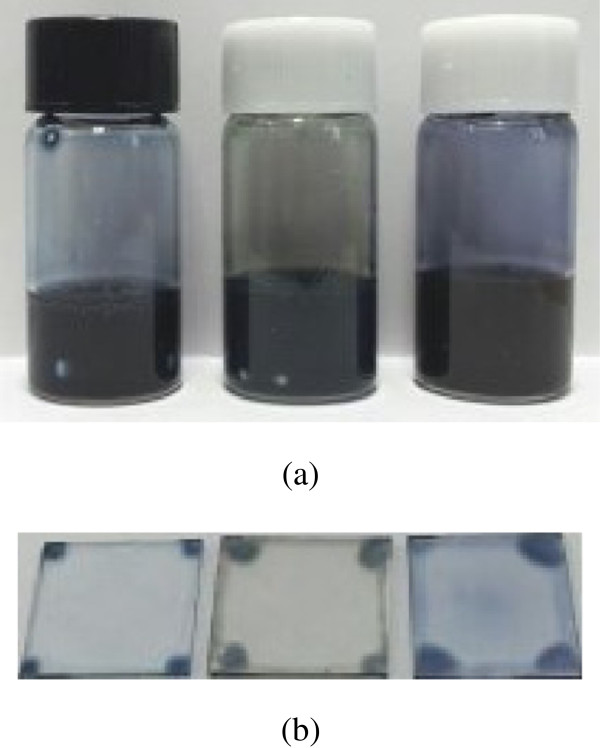
***In situ*-prepared PEDOT:PSS-metal NP solutions (a) and their corresponding spin-coated films on glass substrates.** (**b**) (Pristine PEDOT:PSS, PEDOT:PSS-Ag NPs, and PEDOT:PSS-Au NPs from left to right).

In our new method, we synthesize metal NPs via PEDOT:PSS media. Therefore, there is no need to use a stabilizer or an additional mixing step to prepare metal NPs with a PEDOT:PSS solution. After *in situ* preparation, the color of the mixed solution became pale green (for Ag NPs) or medium purple (for Au NPs) from the original pristine royal blue (for PEDOT:PSS), indicating that the Ag and Au NPs had been successfully synthesized in the PEDOT:PSS media, as shown in Figure 3. The corresponding spin-coated films also showed unique colors due to the SP effects.

**Figure 4:**
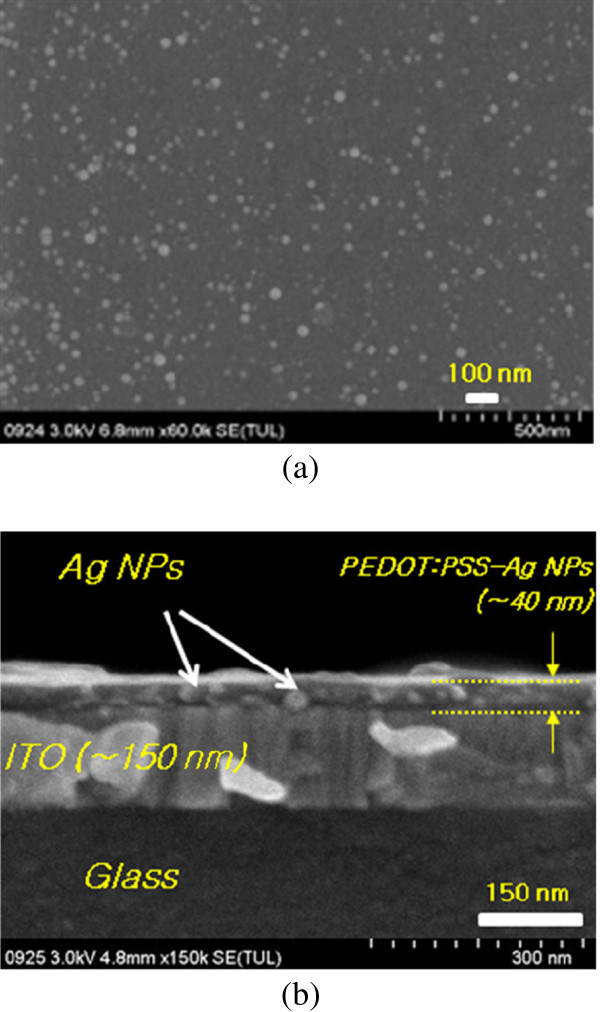
**SEM image of Ag NPs within PEDOT:PSS media and cross-sectional view.** SEM image of (**a**) Ag NPs within PEDOT:PSS media in the drop-casted film state, and (**b**) cross-sectional view of spin-casted PEDOT:PSS-Ag NP thin layer.

Figure 4a shows a scanning electron microscopy (SEM) image of the synthesized Ag NPs. As shown in the figure, the Ag NPs are shapeless or roughly spherical and have average diameters of 20 to approximately 40 nm. From the cross-sectional view of the glass/ITO/PEDOT:PSS-Ag NPs shown in Figure 4b, most NPs are fully embedded into the PEDOT:PSS; therefore, the surface of the PEDOT:PSS appears to have some degree of roughness. Ag NPs generally produce an absorption peak between 400 and 450 nm, and Au NPs have a surface plasmon resonance (SPR) peak between 500 and 600 nm depending on the thickness, shape, and distance of the nanostructures [24]. Because the main absorption range of P3HT is from 400 to 650 nm, Ag and Au NPs are both appropriate materials for improving the performance of OSCs by the SPR effect.

**Figure 5:**
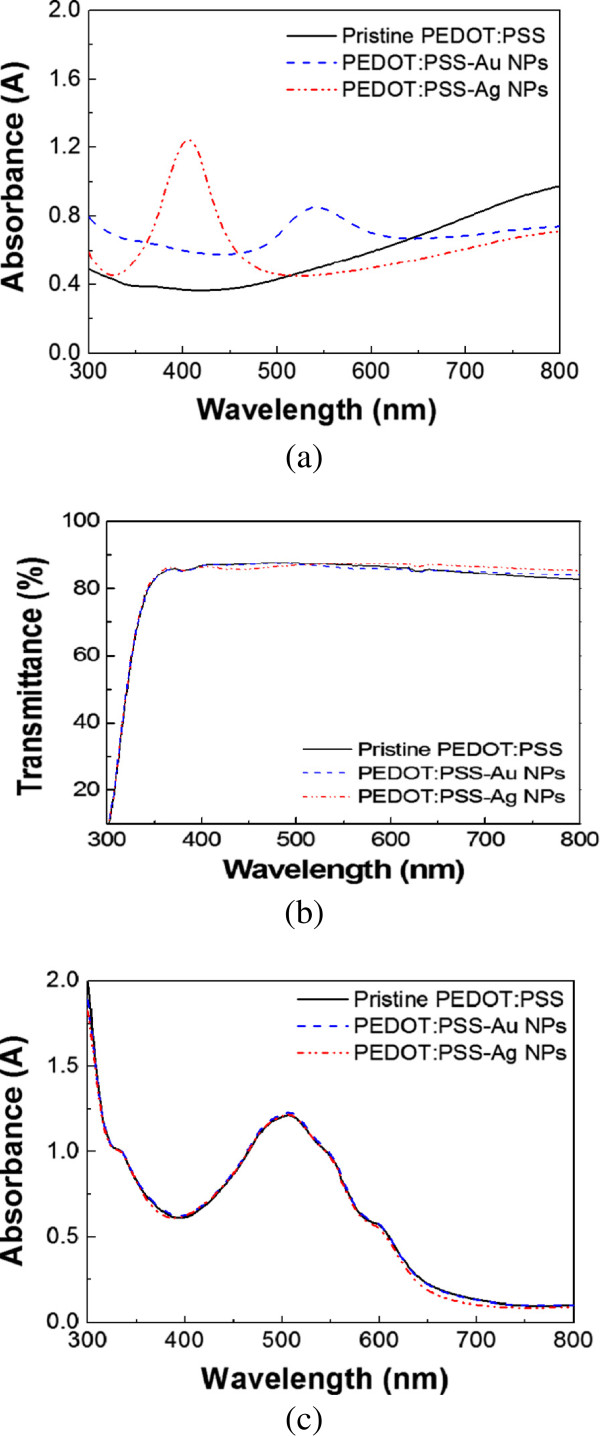
**Optical properties of PEDOT:PSS-metal NPs.** (**a**) UV–vis absorption spectra of the PEDOT:PSS-metal NPs in solutions, (**b**) transmittance spectra of the glass/PEDOT:PSS-metal NPs, and (**c**) absorption spectra of glass/PEDOT:PSS-metal NPs/active layer.

Figure 5a shows the absorption spectra of diluted pristine as well as Ag- and Au NP-incorporated PEDOT:PSS solutions as measured by a PerkinElmer Lambda 750 UV–vis spectrometer (PerkinElmer Inc., Waltham, MA, USA). PEDOT:PSS with Ag NPs shows a SPR peak at about 405 nm, and PEDOT:PSS with Au NPs has a SPR peak at 550 nm. The transmission spectra of the glass/PEDOT:PSS-metal NPs show a slightly decreased region between 400 and 500 nm (Ag NPs) and 500 and 600 nm (Au NPs), which is indicative of SP absorption by metal NPs. However, the contribution of SP absorption in the PEDOT:PSS-metal NPs to the light absorption properties of the photoactive layer is negligible, as shown in Figure 5c, in the wavelength between 400 and 600 nm. In theoretical and experimental analyses found in previous reports, the strong near-field effect around metal NPs by SPR has a certain directionality which is mainly distributed along the lateral direction of the PEDOT:PSS layer and not in a direction vertical to the photoactive layer when the incidence of light is normal to the ITO [20,22]. Because metal NPs are entirely embedded in PEDOT:PSS in our system, the lateral SPR effect by NPs is too weak to affect the light-harvesting activity in the photoactive layer, leading to no obvious enhancement of the absorption spectra in Figure 5c.

Prior to checking the device performance, we measured the sheet resistance of the PEDOT:PSS film and the effect of residual impurities remaining in PEDOT:PSS-metal NPs solution on the device performance. The sheet resistance measured by a four-point probe was slightly reduced for PEDOT:PSS-Au NPs (0.81 MΩ/sq) or increased for PEDOT:PSS-Ag NPs (1.39 MΩ/sq) compared to pristine PEDOT:PSS (1.12 MΩ/sq). We also noted that dissolving each small amounts of NaBH_4_, HAuCl_4,_ or AgNO_3_ into PEDOT:PSS did not affect the device performances (data not shown here). This indicates that the effects of the electrical conductivity and residual impurities in the PEDOT:PSS-metal NPs are not likely to be important factors in our device system.

**Figure 6:**
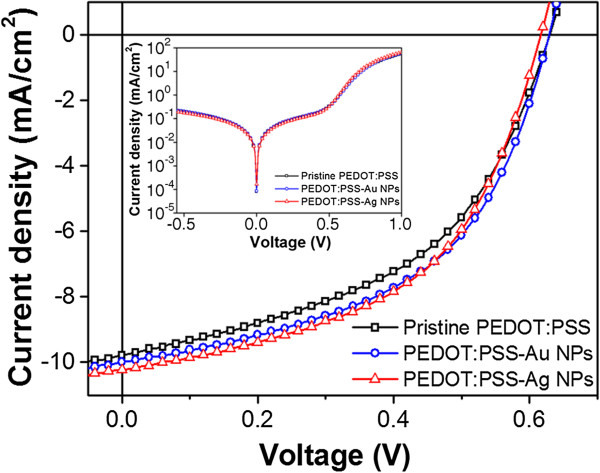
***J*-*V *characteristics of the three devices.**

Figure 6 and Table 1 show the photocurrent density voltages (*JV*) of three different OSC devices measured at 100 mW/cm^2^ (AM 1.5 G) with a Keithley model 2400 source meter (Keithley Instruments Inc., Cleveland, OH, USA) and a Newport 91192 solar simulator system (equipped with a 1 kW xenon arc lamp, by Oriel Instruments, London, UK). The reference device with a pristine PEDOT:PSS buffer layer exhibited an open-circuit voltage (*V*_oc_) of 0.62 V, a short-circuit current density (*J*_sc_) of 9.78 mA/cm^2^, and a fill factor (FF) of 48.7%; thus, the calculated PCE was 2.95%. Considering the enhancement of the *J*_sc_ and FF values, the PCE was improved by 3.2% by applying the *in situ*-prepared PEDOT:PSS-metal NPs buffer layer. From the dark *JV* characteristics in the inset of Figure 6, there is no significant change in the leakage current in both the Au NPs and the Ag NPs, which is an additional evidence of the full coverage of metal NPs with the PEDOT:PSS media. The incorporation of metal NPs into PEDOT:PSS increased both the surface roughness of the PEDOT:PSS layer and the PEDOT:PSS/P3HT:PCBM interface area. It has been reported that an increased surface roughness of the buffer layer as well as an enlarged interface area of PEDOT:PSS/P3HT:PCBM lead to shortening of the hole-extraction route and enhancement of the hole-collection efficiency by the anode [15,22,25]. The reduced series resistance (*R*_s_) from 1.8 to 1.5 Ωcm^2^ in Table 1 is another indicator of the improved hole collection and ultimately, the enhancement of *J*_sc_ and FF.

**Table 1:** Summary of device performances

	***V***_**oc**_**(V)**	***J***_**sc**_**(mA**/**cm**^**2**^**)**	**FF (%)**	**PCE (%)**	***R***_**s**_**(Ωcm**^**2**^**)**
Pristine PEDOT:PSS	0.62	9.78	48.7	2.95	1.8
With Au NPs	0.62	9.99	51.5	3.19	1.5
With Ag NPs	0.61	10.24	51.2	3.20	1.5

## Conclusions

In conclusion, we successfully synthesized composites materials of PEDOT:PSS-Au or Ag NPs by a simple *in situ* preparation method. The synthesized NPs with a size of 20 to approximately 40 nm were well dispersed in PEDOT:PSS media and showed a characteristic absorption peak due to the SPR effect. According to our *J*-*V* and UV–vis findings, the 8% improvement of the PCE is mainly caused by the increased hole collection to the anode due to the increased surface roughness and increased interface area of the buffer layer. Moreover, the optical effects of metal NPs are a minor factor when the metal NPs are embedded fully into the PEDOT:PSS layer.

Our *in situ* method provides an easy solution to stabilize metal NPs dispersed in water-based functional polymers as it does not require the use of an organic capping agent. This can be a suitable means of incorporating metal NPs for the future practical application of organic electronics such as organic memory, organic thin film transistors, and organic solar cells without any change to the conventional device structure or fabrication process.

## Competing interests

The authors declare that they have no competing interests.

## Authors' contributions

SW and JHJ synthesized, characterized, and fabricated the OSC device. HKL and YSH participated in the scientific flow and discussions. SW and YK conceived of the study and participated in its design and coordination. All authors read and approved the final manuscript.
