# Correction: *in situ*-prepared composite materials of PEDOT: PSS buffer layer-metal nanoparticles and their application to organic solar cells

**DOI:** 10.1186/1556-276X-9-506

**Published:** 2014-09-16

**Authors:** Sungho Woo, Jae Hoon Jeong, Hong Kun Lyu, Yoon Soo Han, Youngkyoo Kim

**Affiliations:** 1 Green Energy Research Division, Daegu Gyeongbuk Institute of Science and Technology (DGIST), Daegu 711-873, South Korea; 2 Organic Nanoelectronics Laboratory, Department of Chemical Engineering, Kyungpook National University, Daegu 702-701, South Korea; 3 Department of Advanced Energy Material Science and Engineering, Catholic University of Daegu, Gyeongbuk 712-702, South Korea

## Correction statement

In the Methods section of our published article [1], we mentioned that “we developed a novel *in situ* means of preparing stabilized Au or Ag NPs…”. However, we have recently noticed that the similar process has been reported by Moreno et al. [2]. Hence, we correct the statement in our manuscript by crediting the work by Moreno et al. [2]:

[Before Correction]

“We developed a novel *in situ* means of preparing stabilized Au or Ag NPs by the reduction of chloroauric acid (HAuCl_4_) or silver nitrate (AgNO_3_) with a sodium borohydride (NaBH_4_) solution in the presence of aqueous PEDOT:PSS media”.

[After Correction]

“We used a simple *in situ* procedure of preparing stabilized Au or Ag NPs by the reduction of chloroauric acid (HAuCl_4_) or silver nitrate (AgNO_3_) with a sodium borohydride (NaBH_4_) solution in the presence of aqueous PEDOT:PSS media [2-4]”.
